# Blunt Cardiac Injury Resulting in Avulsion of Septal Myocardium Without Ventricular Septal Defect

**DOI:** 10.7759/cureus.85722

**Published:** 2025-06-10

**Authors:** Kimberly R Ding, Benjamin Noor, Rooqash Ali, Ignacio D Velazquez

**Affiliations:** 1 Internal Medicine, Harbor-UCLA Medical Center, Torrance, USA; 2 Cardiology, Harbor-UCLA Medical Center, Torrance, USA; 3 Neurology, Harbor-UCLA Medical Center, Torrance, USA; 4 Internal Medicine and Cardiology, Harbor-UCLA Medical Center, Torrance, USA

**Keywords:** avulsion, blunt cardiac injury, mitral regurgitation, trauma, ventricular septal defect

## Abstract

An avulsion of the ventricular wall refers to the traumatic tearing or detachment of a portion of the heart’s ventricular wall. We report a case involving a man in his 70s with a remote history of seizures, status post (s/p) craniotomy, and a prior gunshot wound to the abdomen s/p exploratory laparotomy with partial colectomy. He presented after sustaining a blunt chest crush injury when a car rolled and pinned him underneath at a mechanic shop. Prognosis in such cases depends on the severity of the injuries. He was found to have an avulsion of the left ventricular septal myocardium without an associated ventricular septal defect or mitral regurgitation. Brain imaging revealed multivessel territory ischemia. Following a multidisciplinary discussion, the patient was managed conservatively with anticoagulation due to the theoretical risk of thrombus formation, along with serial echocardiographic monitoring. Although he remained cardiovascularly stable at discharge, prolonged encephalopathy with persistent ventilator dependence required transfer to a long-term acute care facility.

## Introduction

Blunt chest trauma refers to a nonpenetrating injury to the chest, typically caused by sudden impact, collision, or compression of the thoracic region. Approximately 20% of such cases involve the heart, resulting in blunt cardiac injury (BCI) [1]. As BCI encompasses a spectrum of outcomes, the literature commonly reports traumatic ventricular septal defects (VSDs) alongside injuries such as valvular damage, rupture of the chordae tendineae or papillary muscles, and aneurysm formation, most often in the context of high-speed motor vehicle accidents requiring surgical intervention [1-5]. Prognosis largely depends on the severity of injury and the timeliness of treatment. For example, isolated myocardial contusions often resolve with monitoring and supportive care, whereas more severe injuries involving rupture or tamponade are associated with high mortality rates, ranging from 20% to 50% [1-5].

This case highlights a BCI resulting from a slow crush injury in an elderly patient, which was ultimately managed conservatively. This mechanism is notable as it challenges the typical association between traumatic VSDs and high-velocity impacts, suggesting that even slow, sustained chest compression can generate significant intrathoracic forces during vulnerable phases of the cardiac cycle. The patient’s hemodynamic stability and absence of valvular dysfunction supported the decision for conservative management, avoiding the need for surgical intervention.

## Case presentation

A man in his 70s with a remote history of seizures and a prior craniotomy, as well as a gunshot wound to the abdomen treated with exploratory laparotomy and partial colectomy over 10 years ago, was brought in following blunt chest trauma. The patient had been working at an auto mechanic shop when a car rolled backward, pinning him underneath for an unknown duration. On arrival to the emergency department, he was alert with a Glasgow Coma Scale (GCS) of 15, presenting with a chest wall deformity resembling pectus excavatum, overlying contusion on the left lower chest, and hemoptysis. He was able to speak in short sentences but was limited by severe pain. Initial resuscitation included 2 L of normal saline (0.9%).

By hour 3, the patient developed persistent hypotension, worsening hypoxia, and a decline in GCS to 8, raising concern for airway protection, and he was subsequently intubated. By hour 10, he required three vasopressors, likely due to cardiogenic shock. A whole-body CT scan revealed multiple facial and rib fractures, flash pulmonary edema, and possible pulmonary hemorrhage.

Investigations

Initial laboratory results showed an elevated troponin-I of 9.995 ng/mL (reference: <0.010 ng/mL), which peaked at 11.312 ng/mL two hours later, and a lactate level of 6 mmol/L (normal: <2.2 mmol/L). The patient maintained adequate urine output throughout his hospital course.

**Figure 1:**
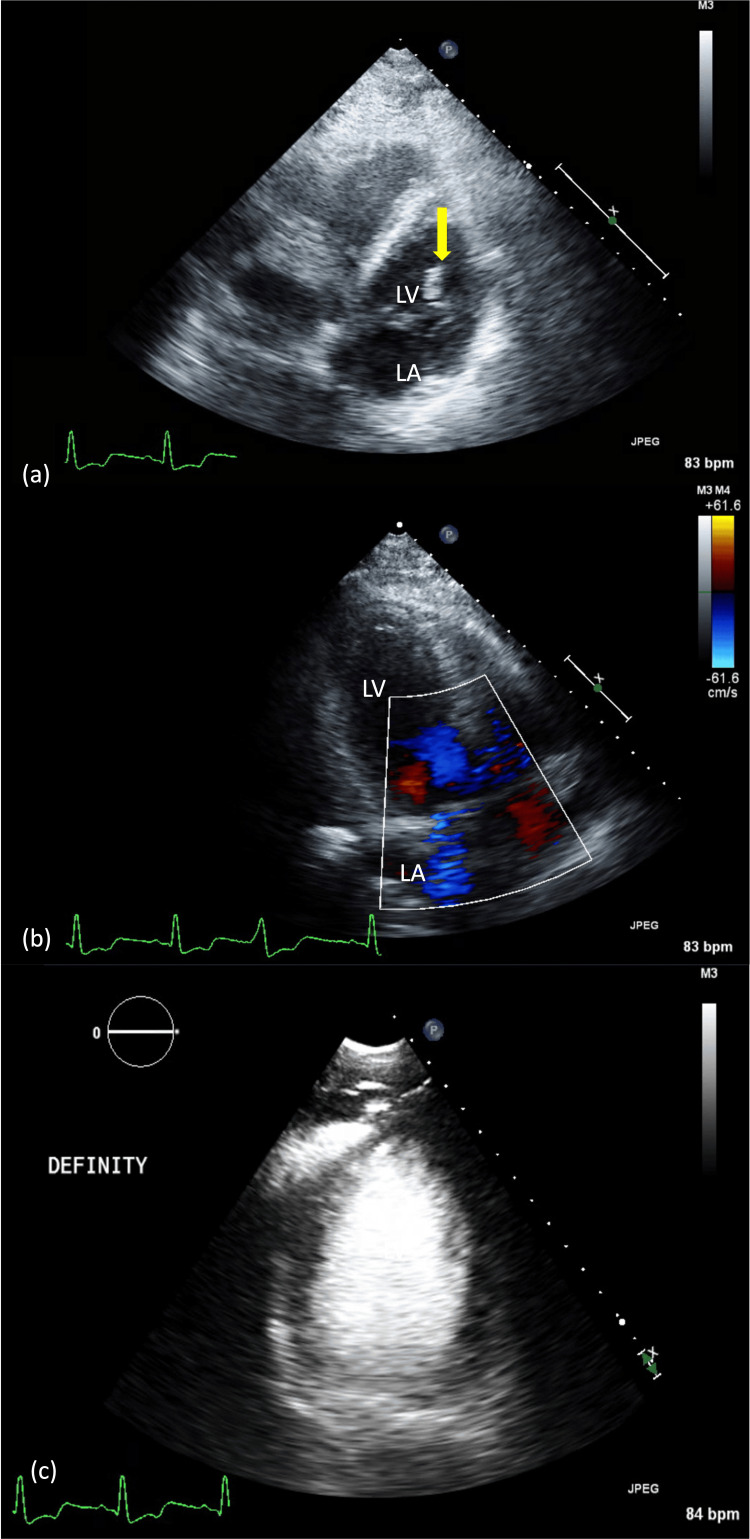
TTE views (a) Modified apical four-chamber view shows a linear echodensity (yellow arrow) within the LV cavity, appearing to be attached to the myocardium. (b) Modified apical three-chamber view demonstrates no evidence of associated MR or VSD. (c) Focused apical four-chamber view with echo contrast confirms the absence of an apical thrombus. LV, left ventricular; MR, mitral regurgitation; TTE, transthoracic echocardiogram; VSD, ventricular septal defect

A transthoracic echocardiogram (TTE) performed on hospital day 2 (Figure 1a) demonstrated an ejection fraction of 40-45%, with a linear, mobile echodensity contiguous with the septum and subvalvular apparatus in the left ventricle (Video 1). There was no significant mitral regurgitation (MR) to suggest complete papillary muscle rupture, nor was there evidence of an apical thrombus on DEFINITY® contrast imaging (Lantheus Medical Imaging, Inc., North Billerica, Massachusetts, United States) (Figure 1b, 1c).

**Video 1:** TTE of apical four-chamber view This TTE recording highlights the echodensity in question, providing better visualization of its contiguous nature consistent with myocardial avulsion. TTE, transthoracic echocardiogram

A transesophageal echocardiogram (TEE) was planned for further evaluation, but due to multiple facial fractures, the procedure was deferred for safety reasons. TEE provides high-resolution, close-range imaging of the left ventricular (LV) septum and adjacent valves, potentially detecting subtle structural injuries that may be missed on TTE.

**Figure 2:**
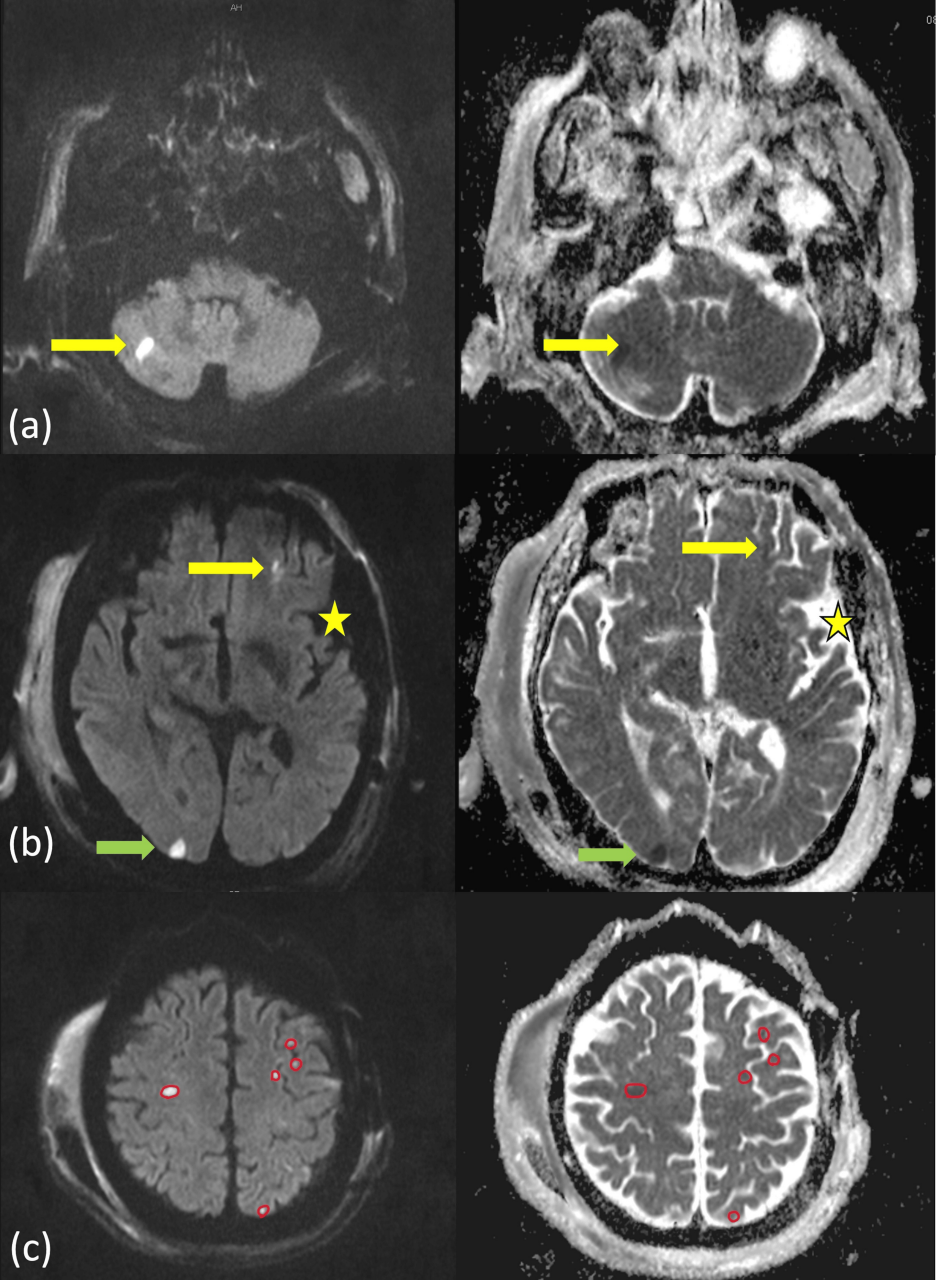
MRI findings on DWI and ADC sequences Axial cuts from DWI (left panel) and ADC (right panel) sequences demonstrate (a) hyperintensities on DWI with corresponding hypointense and some isointense areas on ADC in the cerebellar region (yellow arrows), (b) punctate foci of diffusion restriction at the level of the third ventricle, involving the left frontal territory (yellow arrow) and right occipital lobe (green arrow), along with evidence of a prior left craniotomy (yellow star), and (c) multiple areas of diffusion restriction at the level of the centrum semiovale, involving frontoparietal distributions. These findings indicate acute to subacute strokes in both anterior and posterior circulations, raising concern for a possible cardiogenic source of emboli. ADC, apparent diffusion coefficient; DWI, diffusion-weighted imaging

In the meantime, due to concern for seizures, a brain MRI without contrast was performed. This revealed diffusion restriction on diffusion-weighted imaging in the right occipital lobe and bilateral cerebellum, with corresponding hypointense areas on apparent diffusion coefficient sequences - a pattern highly suggestive of cardioembolic stroke (Figure 2a-2c). 

By hospital day 8, the patient was evaluated and cleared by the neurosurgery and plastic surgery teams to undergo TEE. This was done to further assess the LV echodensity and to provide risk stratification for possible secondary stroke prevention and anticoagulation therapy.

**Figure 3:**
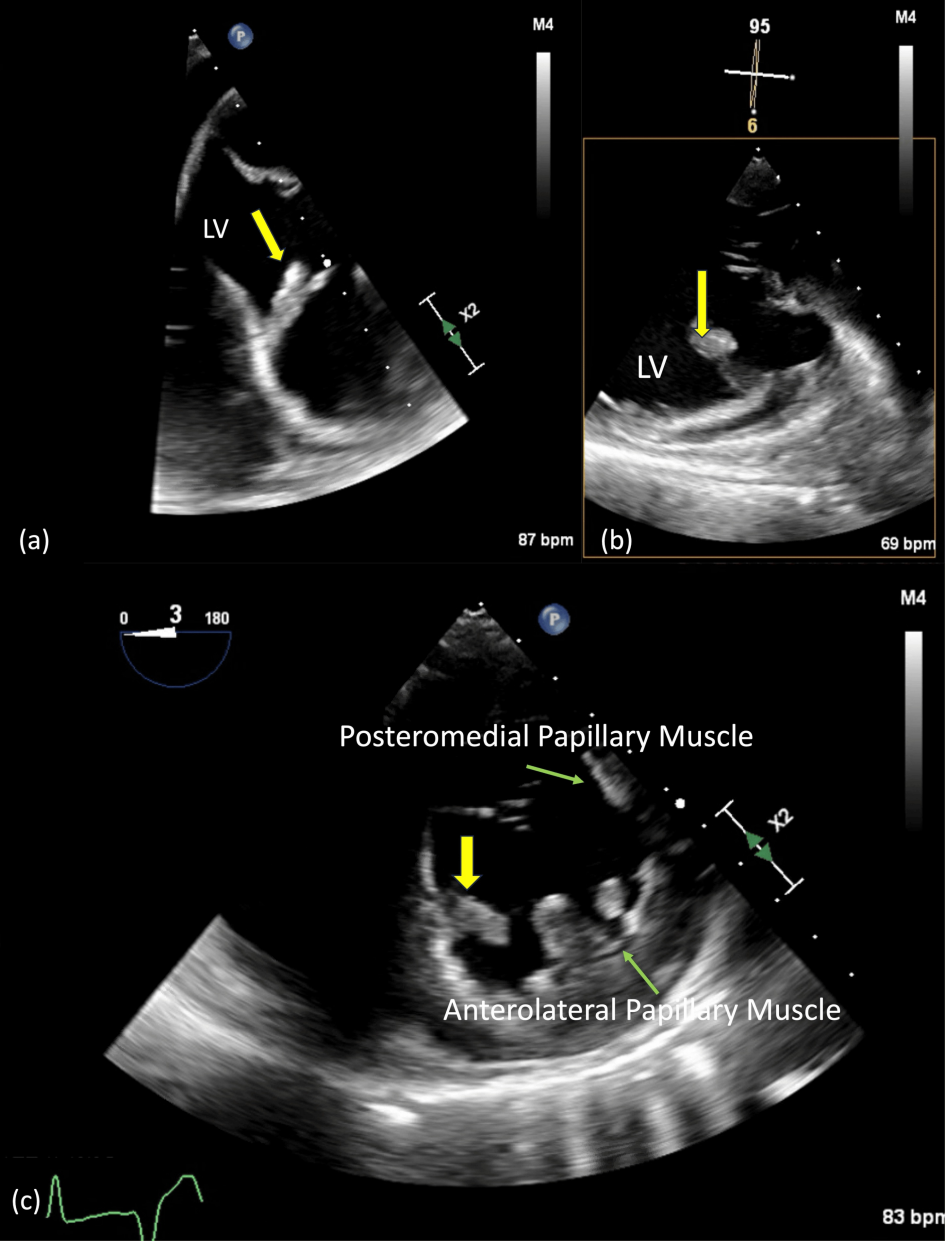
TEE views (a) Modified mid-esophageal, (b) transgastric long-axis, and (c) transgastric short-axis views of the LV cavity reveal a 17 × 8 mm segment of avulsed myocardium (yellow arrows) tethered by a stalk to the inferoseptal wall. Papillary muscle architecture appears preserved (green arrows). LV, left ventricular; TEE, transesophageal echocardiogram

The TEE showed a 17 × 8 mm echodensity attached by a stalk to the ventricular septal wall, with no associated VSD (Figure 3a-3c). The mitral valve chordae and papillary muscles remained intact, with no evidence of rupture. Additionally, there was no thrombus identified in the left atrial appendage or the left ventricle.

Two more limited TTEs were performed to exclude VSD, with the last surveillance imaging done on hospital day 24.

Differential diagnosis

Given the clinical context of BCI and the size and location of the echodensity contiguous with the interventricular septum, the findings were most consistent with septal myocardial avulsion without evidence of a VSD. Papillary muscle rupture was also considered; however, both the initial and follow-up TTEs showed no significant MR, no flail leaflet with systolic prolapse into the left atrium, and no mobile mass attached to the chordae tendineae. The absence of MR supports the diagnosis of isolated septal avulsion, indicating preservation of the mitral valve apparatus, including the papillary muscles and chordae tendineae. This distinction is clinically important, as more extensive myocardial rupture usually involves valvular disruption and resultant regurgitation.

The follow-up TEE also confirmed preserved papillary muscles. Although the patient was in shock, the cardiac index remained stable at around 2.4 L/min/m². Other differential diagnoses included LV thrombus [6-8], which typically forms within or adjacent to the LV apex and is associated with wall motion abnormalities, findings inconsistent with this patient’s echocardiogram. Infectious causes, such as interventricular abscess, were considered but were unlikely given the clinical context (no fevers, no ECG abnormalities suggestive of heart block) and absence of heterogeneous enhancement on imaging [9].

Other possibilities included cardiac tumors such as fibroma or fibroelastoma; however, cardiac tumors are exceedingly rare in adults (prevalence approximately 0.02%) [10]. Fibromas are typically larger, averaging around 50 mm, and usually present as well-circumscribed intramyocardial lesions rather than protrusions [11]. Fibroelastomas, on the other hand, tend to be pedunculated and attached to valves, more commonly the aortic valve than the mitral [6,11].

We hypothesize that the cardiac septal avulsion served as a nidus for thrombus formation, predisposing the patient to cardioembolic events. The abnormal septal integrity and associated turbulent flow may have promoted local clot formation, which subsequently embolized to the cerebral circulation. This hypothesis is supported by MRI findings showing acute ischemic infarcts in multiple vascular territories, a pattern characteristic of cardioembolic stroke.

Treatment

Through multidisciplinary collaboration among cardiology, neurology, and the surgical intensive care unit teams, the patient was started on a heparin infusion targeting an anti-Xa level of 0.30-0.70 IU/mL on hospital day 4. A surveillance non-contrast head CT performed on day 11 ruled out hemorrhagic transformation of the previously identified ischemic infarcts. Repeat imaging is critical in this setting, as anticoagulation carries an inherent risk of intracranial bleeding, particularly during the subacute phase of stroke recovery. After confirming radiographic stability, the patient was safely transitioned from intravenous heparin to oral anticoagulation with rivaroxaban 15 mg twice daily on hospital day 16.

Outcome and follow-up

The patient had no further imaging evidence of new ischemic events and remained hemodynamically stable without signs of cardiogenic shock. However, he continued to experience significant respiratory distress. Although he was briefly extubated, he became progressively hypoxic with increasing respiratory secretions that could not be effectively cleared despite appropriate pulmonary hygiene and supportive care. The patient remained afebrile, and serial chest radiographs showed no new consolidations, with stable bilateral pleural effusions unchanged from prior imaging during the extubation period.

Due to prolonged respiratory failure, he ultimately required tracheostomy and gastrostomy tube placement. Rehabilitation planning was limited given uncertainty about his response to future spontaneous breathing trials, and his overall prognosis was considered guarded. He was discharged on hospital day 30 to a long-term acute care facility, with plans for outpatient follow-up, including a surveillance TTE in 4-6 weeks to monitor for progression of the septal avulsion. Anticoagulation with rivaroxaban 15 mg twice daily was continued pending reassessment by outpatient neurology.

## Discussion

Our case highlights a unique presentation and clinical course involving a rare cardiac injury following trauma. The injury mechanism was a slow, blunt crush trauma, which is a less commonly reported cause of cardiac damage. Traumatic VSD is thought to result from substantial force applied to the chest wall during isovolumic contraction - a phase when the ventricles are full and the atrioventricular valves are closed, creating a vulnerable moment for structural failure. Unlike previously reported cases involving full-thickness VSD or associated valvular disruption, our patient exhibited isolated septal avulsion without papillary muscle rupture, flail leaflets, or significant MR. The absence of hemodynamic instability or progressive valvular dysfunction allowed for conservative management without surgical intervention.

Unexpectedly, incidental MRI findings revealed multifocal ischemic strokes across multiple vascular territories. The patient had no history of atrial fibrillation, no major blood pressure fluctuations, normal lipid levels, and no evidence of atherosclerotic large vessel disease. The National Institutes of Health Stroke Scale (NIHSS) evaluation was not performed on admission due to the absence of focal neurological deficits and was precluded after the infarcts were discovered, as the patient was already intubated. However, the lack of an NIHSS score did not change clinical judgment. The decision to anticoagulate was based on imaging characteristics suggestive of embolic stroke and a plausible cardiac source. The imaging pattern aligned with a cardioembolic etiology, raising the exposed septal endocardium as a potential site for thrombus formation and embolism. Given the theoretical risk of clot propagation at the site of myocardial disruption, and in the absence of other clear embolic sources, we started intravenous unfractionated heparin anticoagulation, targeting standard anti-Factor Xa levels (0.3-0.7 IU/mL).

This case highlights an important gap in current literature. While existing AHA/ASA stroke guidelines recommend thorough secondary risk stratification - including rhythm monitoring and echocardiographic evaluation for patients with cryptogenic or embolic-appearing strokes - they do not recognize traumatic myocardial injuries such as septal avulsion as potential cardioembolic sources [12,13]. Most literature on cardiac trauma focuses on surgical repair of septal or valvular rupture, without established guidance on anticoagulation for myocardial avulsion [1,5]. Our case raises the possibility that rare structural cardiac disruptions, especially those caused by trauma, may be an underrecognized source of cardioembolic stroke. Further research is needed to clarify when anticoagulation is appropriate in these cases and to inform future guidelines.

## Conclusions

This case underscores the diagnostic challenges posed by rare myocardial injuries after blunt chest trauma, particularly when findings are subtle or atypical. It emphasizes the importance of integrating echocardiographic data, serial imaging, and clinical context to differentiate structural injuries like septal avulsion from more common sequelae such as papillary muscle rupture or valvular disruption. Careful interpretation of imaging, especially in the absence of classic signs like MR or flail leaflet, was crucial in guiding conservative management and anticoagulation decisions in this complex scenario. The authors acknowledge the lack of long-term follow-up and outcome data for this case.
